# The Wnt/β-catenin pathway in human fibrotic-like diseases and its eligibility as a therapeutic target

**DOI:** 10.1186/s40591-015-0038-2

**Published:** 2015-01-30

**Authors:** Maria Vittoria Enzo, Marco Rastrelli, Carlo Riccardo Rossi, Uros Hladnik, Daniela Segat

**Affiliations:** Genetics Unit, “Mauro Baschirotto” Institute for Rare Diseases, Via B. Bizio, 1- 36023 Vicenza, Italy; Melanoma and Sarcoma Unit, Veneto Institute of Oncology, IOV-IRCSS, Via Gattamelata, 64-35128 Padua, Italy; Department of Surgical Oncological and Gastroenterological Science, University of Padua, Via Giustiniani, 2- 35124 Padua, Italy

**Keywords:** Wnt pathway, Adenomatous polyposis coli, Beta-catenin, Inflammatory factors, Fibrosis, Desmoid-like fibromatosis

## Abstract

The canonical Wnt signaling pathway is involved in a variety of biological processes like cell proliferation, cell polarity, and cell fate determination. This pathway has been extensively investigated as its deregulation is linked to different diseases, including various types of cancer, skeletal defects, birth defect disorders (including neural tube defects), metabolic diseases, neurodegenerative disorders and several fibrotic diseases like desmoid tumors. In the "on state", beta-catenin, the key effector of Wnt signaling, enters the nucleus where it binds to the members of the TCF-LEF family of transcription factors and exerts its effect on gene transcription. Disease development can be caused by direct or indirect alterations of the Wnt/β-catenin signaling.

In the first case germline or somatic mutations of the Wnt components are associated to several diseases such as the familial adenomatous polyposis (FAP) - caused by germline mutations of the tumor suppressor adenomatous polyposis coli gene (*APC*) - and the desmoid-like fibromatosis, a sporadic tumor associated with somatic mutations of the β-catenin gene (*CTNNB1).*

In the second case, epigenetic modifications and microenvironmental factors have been demonstrated to play a key role in Wnt pathway activation. The natural autocrine Wnt signaling acts through agonists and antagonists competing for the Wnt receptors. Anomalies in this regulation, whichever is their etiology, are an important part in the pathogenesis of Wnt pathway linked diseases. An example is promoter hypermethylation of Wnt antagonists, such as SFRPs, that causes gene silencing preventing their function and consequently leading to the activation of the Wnt pathway. Microenvironmental factors, such as the extracellular matrix, growth factors and inflammatory mediators, represent another type of indirect mechanism that influence Wnt pathway activation. A favorable microenvironment can lead to aberrant fibroblasts activation and accumulation of ECM proteins with subsequent tissue fibrosis that can evolve in fibrotic disease or tumor.

Since the development and progression of several diseases is the outcome of the Wnt pathway cross-talk with other signaling pathways and inflammatory factors, it is important to consider not only direct inhibitors of the Wnt signaling pathway but also inhibitors of microenvironmental factors as promising therapeutic approaches for several tumors of fibrotic origin.

## Introduction

The Wnt signaling pathway is involved in several essential biological processes in both embryonic development and in adult cell maintenance and regeneration.

The canonical or Wnt/β-catenin dependent pathway controls key developmental gene expression programs by modulating the amount of β-catenin through regulating its degradation or accumulation and its translocation from the adherens junction and cytoplasm to the nucleus. In the absence of a Wnt signal, the cytoplasmic β-catenin is tightly maintained at a low level by a multiprotein destruction complex consisting of Axin, the adenomatous polyposis coli protein (APC), casein kinase 1α (CK1α), and Glycogen Synthase Kinase 3β (GSK3β). The complex phosphorylates cytoplasmic β-catenin leading to its degradation by the ubiquitin-proteasomal system. The continuous elimination of β-catenin prevents its accumulation in the cytoplasm and the consequent translocation into the nucleus. In the presence of a Wnt signal, the destruction complex is disassembled leading to an increment of β-catenin levels and allowing its translocation into the nucleus where it activates Wnt target gene expression. The aberrant regulation of the Wnt/β-catenin pathway plays a role in the pathogenesis of several diseases including cancer, birth defect disorder, skeletal diseases, and fibrotic diseases. For this reason Wnt/β-catenin signaling is tightly regulated and kept under strict control at different levels of the Wnt cascade. Wnt activation is temporally and spatially tuned by autocrine Wnt signaling that is associated with extracellular Wnt agonists and antagonists. The agonists activate the Wnt cascade while the antagonists inhibit Wnt signaling at the level of ligand/receptor [1,2]. However, Wnt/β-catenin signaling deregulation can occur via several mechanisms. In particular, germline mutations of the tumor suppressor gene *APC* are associated with familial adenomatous polyposis (FAP), and somatic mutations of the β-catenin gene (*CTNNB1)* are associated with sporadic desmoid tumors. In the first case the disease is caused by a transmissible genetic defect, in the second case the pathology is linked to a somatic mutation that makes β-catenin unable to be completely phosphorylated and degraded.

Wnt/β-catenin signaling can be also indirectly altered by epigenetic modifications that cause silencing of Wnt endogenous brakes, and by the effect of microenvironmental factors, such as the extracellular matrix, hormones and growth factors. Of particular interest is the involvement of inflammatory factors in the modulation of the Wnt/β-catenin pathway and its association with fibrotic disease as well as tumor development.

Either direct or indirect Wnt pathway alterations can cause an increase of β-catenin levels and its accumulation into the nucleus, activating the signaling cascade. The cross-talk between these extracellular stimuli and intracellular signals highlights the complex interaction of the numerous factors involved in the development of the Wnt pathway linked pathologies and are well represented in fibrotic disease and in particular in the sporadic desmoid tumors.

Many studies describe the use of small synthetic molecules for inhibiting the β-catenin as therapeutic approach. Among these, there are molecules that target the interaction of β-catenin with co-activators disabling the formation of an active transcriptional complex. Recently GSK3β inhibitors have been described as promising drugs for several pathologies such as diabetes, stroke, mood disorders, inflammation, and Alzheimer’s disease. The use of specific inhibitors of the Wnt signaling molecules or/and inhibitors of other signaling pathways associated to β-catenin pathway may help to find the key steps of the different pathologies linked to the Wnt pathway.

## Review

### Wnt pathway

The Wnt pathway is one of the evolutionarily-conserved cell signaling pathways used both during embryogenesis and in developed organism’s homeostasis to regulate cell proliferation, cell polarity, and cell fate determination [3-6]. The extracellular Wnt signal stimulates several intracellular signal transduction cascades, including the non-canonical or β-catenin-independent pathways and the canonical or β-catenin dependent pathway [7].

### Non-canonical pathway

The non-canonical Wnt pathways, defined as Wnt- or Frizzled-mediated (Fzd) signaling independent of β-catenin transcriptional activity [8], are diverse and include the Wnt polarity, Wnt-Ca^2+^, and Wnt-atypical protein kinase C pathways. These pathways have been reported to contribute to developmental processes such as planar cell polarity (PCP), convergent extension movements during gastrulation, neuronal and epithelial cell migration [8-13].

Wnt/Ca^2+^ signaling, in particular, activates heterotrimeric G proteins that stimulate phospholipase C (PLC). The signaling activation results in intracellular Ca^2+^ mobilization with activation of Ca^2+^-dependent effectors that include protein kinase C (*PKC*), calcium calmodulin mediated kinase II (*CAMKII*), and calcineurin [14].

### Canonical pathway

The canonical pathway is the most studied Wnt signaling pathway as it is involved in a variety of biological processes and integrates signals from other cellular pathways. It controls different processes throughout embryonic development, such as stem cell pluripotency, cell proliferation, differentiation, and cell migration. In adult cells, Wnt signaling contributes to maintain somatic stem cells, regulates cell fate decisions and it is involved in tissue regenerative processes following injury [15].

**Figure 1:**
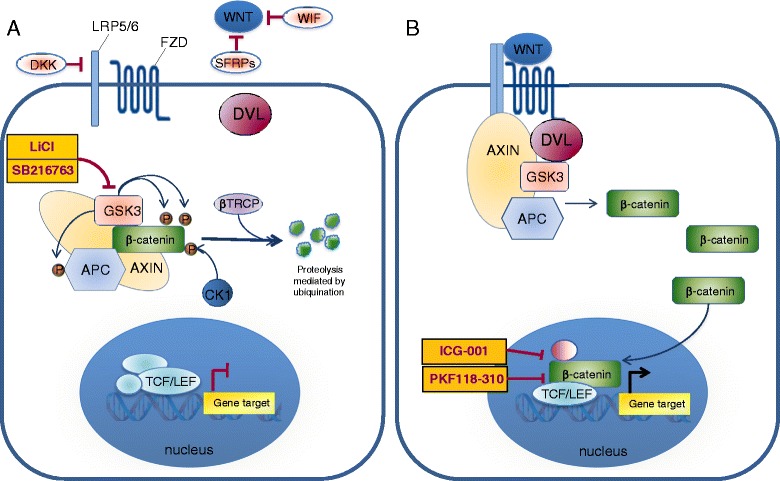
**The canonical Wnt/β-catenin pathway. A)** In absence of Wnt signal the destruction complex, formed by the scaffold Axin, APC and GSK3, phosphorilates (P) β-catenin that is then ubiquitinated and degraded via proteasome. In this state the expression of the gene target is repressed. **B)** In presence of Wnt ligand, the receptor Fzd and the co-receptor LRP5/6 transduce the signal activating Dvl. The destruction complex is inhibited and β-catenin accumulates in the cytoplasm and it translocates into the nucleus. It promotes the target gene expression by binding TCF/LEF and other co-activators. To date several studies identified small molecules (some of these are indicated in the yellow boxes) that can directly inhibit specific components of the Wnt pathway. APC = adenomatous polyposis coli; GSK3 = glycogen synthase kinase; TCF = T cell factor; Fzd = Frizzled receptor; Dvl = Dishevelled.

The hallmark of the canonical Wnt pathway is transcriptional activation by β-catenin. The pathway regulates the amount of β-catenin through its degradation or its accumulation and translocation from the adherens junction and cytoplasm into the nucleus. In this way it controls key developmental gene expression programs [7,16,17]. In the absence of Wnt signaling, cytoplasmic β-catenin is constantly degraded by the ubiquitin–proteasome system. This negative regulation involves the multiprotein complex, composed of Axin, adenomatous polyposis coli (APC), casein kinase 1 (CK1), protein phosphatase 2A (PP2A), and glycogen synthase kinase 3β (GSK3β) [18,19]. Axin interacts with the different components of the complex and coordinates sequential phosphorylation of β-catenin. Initially CK1α phosphorylates β-catenin at serine 45 which enables the phosphorylation performed by GSK3β at threonine 41, serine 37, and serine 33 (Figure 1A) [20]. Subsequently, phosphorylation of APC by CK1α and GSK3β leads to an increased affinity between APC and β-catenin triggering a transfer of β-catenin from Axin to APC and to β-Trcp (β-transducin-repeat-containing protein) [21], an E3 ubiquitin ligase subunit that carries out ubiquitination of β-catenin for the proteasome destruction [16,18,22,23].

The Wnt/β-catenin pathway is activated when specific extracellular molecules, Wnt ligands, bind to a receptor complex consisting of a seven-pass transmembrane Frizzled (Fzd) receptor and its co-receptor, low-density lipoprotein receptor related protein 6 (LRP6) or its close relative LRP5. The activated receptors recruit the scaffolding protein Dishevelled (Dvl), which leads to LRP5/6 phosphorylation, mediated by either CK1γ or GSK3β. These events trigger the translocation of Axin to the membrane where it binds to a conserved sequence in the cytoplasmic tail of LRP5/6 [24,25]. Consequently, the APC/Axin/GSK3β complex is destabilized and β-catenin is released allowing it to translocate to the nucleus by a mechanism that is still poorly understood (Figure 1B) [26,27]. In the nucleus, β-catenin binds to the members of the lymphoid enhancer factor T cell (TCF/LEF) DNA-binding transcription factors and induces the expression of downstream targets including c-Myc, cyclin D1, the matrix metalloproteinase MMP-7, the Ets family transcription factor PEA3 and Axin2 [26,28]. In the absence of the Wnt signal, TCF/LEF factors bind DNA at Wnt-responsive genes and interact with other factors (e.g. Groucho, histone deacetylase) to repress gene transcription.

## Ligands and the main constituents of the Wnt/β-catenin pathway

The Wnt/β-catenin pathway’s complexity derives from the high number of ligands and receptors involved in Wnt signal transduction that can elicit a variety of intracellular responses [10,29].

### Wnt ligands

Wnt ligands comprise a large family of 19 highly conserved cysteine-rich proteins of approximately 350–400 amino acids that contain an N-terminal signal peptide for secretion [16].

Wnt ligands are involved in both the canonical and the non-canonical pathways. Traditionally some ligands (WNT1, WNT3a, and WNT8) have been classified as canonical ligands and others (WNT4, WNT5a, and WNT11) as non-canonical ligands but this classification is now viewed as obsolete. Single Wnt ligands can be involved in multiple intracellular cascades and activate both types of pathways with opposing outcomes. The Wnt outcome depends on the receptor status and on the cellular and microenvironmental context [30,31].

### Receptor and co-receptor: Frizzled and LRP

The activation of Wnt/β-catenin signaling requires the cooperation and the aggregation of two types of transmembrane receptors: the Frizzled (Fzd) seven-pass transmembrane G-protein-coupled receptors [32] and the LRP5 and LRP6 [18]. The binding site for Wnt ligands is the extracellular cysteine-rich domain (CRD) that is well conserved between Fzd members [33]. The intracellular C-domain shows sequence diversity among Fzds but a KTxxxW domain is associated with Wnt/β-catenin transduction [31,34,35] and most of Fzd receptors can activate β-catenin signaling [32,36]. In addition to the Fzd-LRP5/6 heterodimerization, Wnt ligands induce LRP5/6 dimerization/oligomerization [26,37] that seems crucial for the canonical pathway activation [16,38]. The ectodomain of LRP5/6 is composed of three LDL repeats (LDLR) and four β-propeller/epidermal growth factor (EGF) repeats (E1-4) that are the binding domain of canonical Wnt ligands and canonical pathway inhibitor Dkk1 [33,38,39]. Chen et al. demonstrated that the receptor complex is maintained in an inactive state when LRP5/6 associates with Fzd. When a Wnt ligand binds to LRP5/6 and Fzd, it is believed to induce a conformational change leading the LRP5/6 dimerization necessary for normal signal transduction [40].

### Dvl

Dishevelled (Dvl) proteins are multifunctional intracellular proteins involved in both canonical and non-canonical pathways and have numerous putative binding partners [41]. In mammals there are three isoforms, Dvl-1, 2, and 3, with a modular structure that contains four distinct domains, a DIX, a PDZ and a DEP domain followed by a C-terminal domain (CTD) [42,43]. The DIX and PDZ domains mediate canonical WNT signaling while the PDZ and DEP domains participate in non-canonical pathways. This suggests that Dvl might function as molecular switch regulated by other extracellular signals [41,44]. Indeed Dvl functions are modulated by several phosphorylation sites that are targets of specific kinases and phosphatases [43,45,46].

### Axin

Axin is a scaffold protein that acts as a constitutive negative regulator of Wnt signaling by forming a complex with β-catenin, APC, and GSK3β. In particular, this function is carried out by the Axin C-terminal DIX domain (DAX domain) [47-49]. The promotion of rapid and reversible homotypic DAX-DAX polymerization [50] allows the assembly of a dynamic interaction platform that increases the binding affinity for other components such as APC and GSK3β [51]. The Axin-DAX domain can also interact with Dvl-DIX domain forming heterotypic Axin-Dvl interactions: this heteropolymerization switches the Wnt/β-catenin state to being active [52,53]. Axin has another structural domain in its N-terminus (the RGS domain), through which it binds directly to APC [51,54,55]. Axin can be phosphorylated by GSK3 and CK1, and this is believed to increase its association with β-catenin. On the other hand, two serine/threonine phosphatases (PP1 and PP2A) hinder the action of GSK3 and CK1 in the Axin complex reducing the β-catenin degradation. In particular, PP1 dephosphorylates Axin and promotes the disassembly of the Axin complex [16,56].

### APC

*APC* is a tumor suppressor gene located on the long arm of chromosome 5 (5q21). APC has multiple domains that mediate oligomerization as well as binding to a variety of other proteins [57], which have an important role in cell adhesion, signal transduction and transcriptional activation [58]. APC is indispensable for Axin’s activity in assembling the destruction complex [51]. APC may cluster multiple Axin molecules directly, through its multiple Axin-binding sites [55], or indirectly through additional factors (such as CtBP) [59]. Mendoza *et al*. speculated that APC competes with Dvl for association with Axin, displacing it from Axin protein complex. Wnt signaling may overcome the competition between APC and Dvl for their binding to Axin, allowing simultaneous interaction of all three proteins [37,51,60,61].

APC can be phosphorylated by CK1/GSK3 increasing its affinity for the same β-catenin domain as Axin, suggesting the role of APC in removing the phosphorylated β-catenin molecules from the complex [20,22,62]. Another study suggested that APC protects β-catenin from dephosphorylation by PP2A thereby enhancing β-catenin phosphorylation/degradation [16,56].

### CK1

CK1 is a monomeric serine/threonine kinase involved in many different cell functions. There are seven members with high homology: α, β, γ1, γ2, γ3, δ, and ε. Each isoform is involved in different steps of Wnt pathway, with different effects. CKIα is the kinase that first phosphorylates β-catenin at S45, preparing the molecule for the following phosphorylations by GSK3β [46,63,64]. CKIε promotes the activation of Wnt pathway. It phosphorylates Dvl on multiple sites enhancing the binding of GSK3-binding protein/Frat (GBP/Frat) to Dvl [46,65]. CKIε also phosphorylates TCF3 increasing its affinity for β-catenin. CKIγ is anchored on the plasma membrane and it interacts with LRP6 [46,66].

### GSK3β

GSK3β is a serine/threonine kinase that is highly conserved from yeast to mammals. In mammals two distinct genes encode two GSK3 isoforms, α (51 kDa) and β (47 kDa), which share 97% amino acid sequence identity within their catalytic domains. The two GSK3 isoforms are ubiquitously expressed and they are involved in a wide variety of essential biological processes such as tissue patterning, glucose metabolism, apoptosis, stem cell homeostasis, and cell cycle regulation [67]. GSK3 has over 40 known direct substrates, and regulates many signaling pathways including the Wnt, MAPK/ERK, BMP, mTOR, and insulin pathways [68,69]. In Wnt signaling, GSK3β is recruited to a multiprotein complex via interaction with Axin, where it phosphorylates β-catenin, marking it for ubiquitination and destruction. Quantitative analysis suggests that the interaction of GSK3β with the Axin enhances phosphorylation of β-catenin by >20000-fold [70]. GSK3 has been proposed to play important roles in human disorders such as bipolar disorder, schizophrenia, Alzheimer disease. It also contributes to neoplastic transformation as it belongs to both the canonical Wnt/β-catenin and the PI3K/Akt signaling systems, two major pathways often dysregulated in cancer [71]. However, to date mutations of GSK3β have not been found in human cancer [72].

### CTNNB1 β-catenin

β-catenin has a dual role in the cells: (a) it is a structural protein, stabilizing cell-cell adhesions, which are essential for normal cell physiology and tissue architecture [73,74]; (b) it is the key mediator of canonical Wnt signal transduction from membrane to nucleus, where it operates as a transcriptional co-activator of the T cell factor (TCF) family of DNA-binding proteins [75]. β-catenin provides a direct connection between extracellular signals, gene transcription and cell cycle control [29,73].

β-catenin protein has three domains: the N-terminal domain, the armadillo domain consisting of 12 armadillo repeats (residues 141–664), and a C-terminal domain. The positively charged armadillo (Arm) repeat is the binding site for most β-catenin binding partners [76,77]. Local charge alterations of β-catenin through phosphorylation at multiple sites have the ability to regulate its affinity to specific protein partners. Phosphorylation at the C-terminal domain decreases the binding of β-catenin to the cadherin adhesion complex, while the N-terminal domain is the site of GSK3 and CK1 phosphorylation which is recognized by the β-TrCP ubiquitin ligase for the β-catenin degradation [78,79].

### Endogenous inhibitors of Wnt

Wnt/β-catenin signaling is endogenously regulated by secreted proteins that antagonize the Wnt ligands and act at the cell surface level in order to inhibit the pathway [80]. Among these, there are secreted frizzled-related proteins (sFRP) and Wnt inhibitor proteins (WIF) that inhibit the interaction between Wnt and its receptors. Another inhibitor, the Dickkopf related protein 1 (DKK-1), is a ligand for the Wnt coreceptors LRP5/6 [81]. DKK-1 antagonizes LRP6 function by disrupting Fzd-LRP6 complex or by interacting with LRP6 and consequently promoting its internalization and degradation (Figure 1A) [82].

## Role of the Wnt pathway in human pathology

Deregulations of the Wnt pathway are linked to several human diseases comprising various types of cancer (including skin, breast and colon cancers), skeletal defects, birth defect disorders (including neural tube defects), fibrotic diseases, metabolic diseases, neurodegenerative disorders and others [7,83]. Several causes can lead to alterations in the Wnt pathway including germline and somatic mutations, epigenetic modifications as well as microenvironmental factors.

### Wnt pathway and genetic disorders

The Wnt pathway has been extensively investigated for its involvement in many types of cancer [28]. Several studies with experimental models demonstrated that a high level of β-catenin activity is required for tumor initiation [70]. In particular, colorectal cancer, desmoid tumor, gastric cancer, melanoma, hepatocellular, prostate, thyroid, ovarian, endometrial cancer, and some subsets of breast cancers harbour β-catenin-stabilizing mutations, including germline *APC* gene and somatic *CTNNB1* gene mutations [30,72,75]. Genetic alterations of *Axin2* has been described in adrenocortical carcinoma [84], hepatocellular carcinoma and it may predispose to colorectal cancer [80,85]. Patients with distinct types of hereditary high bone mass diseases were found to carry mutations in the LRP5 extracellular domain, while mutations in *LRP6* are linked to hereditary disorders as osteoporosis, coronary artery disease, and metabolic syndrome [80]. Mutations in *LRP5* and *TCF7L2* genes may lead to the development of obesity and mellitus diabetes [86,87].

#### *APC* gene mutations

The association between colon cancer and the aberrant regulation of the Wnt pathway has been known since the identification of alterations of chromosome 5q as an early event in the carcinogenic process for hereditary colon tumors (Familial Adenomatous Polyposis, FAP), and the discovery, through different linkage studies, of the *APC* gene at this chromosomal site [88,89].

FAP is a colon cancer predisposition syndrome, which is inherited in an autosomal dominant manner. Clinical diagnosis of FAP can be made when more than 100 adenomatous polyps are identified in the colorectum. FAP patients present not only colorectal adenomas but also various extracolonic manifestations, including desmoid tumors, osteomas, dental abnormalities, congenital hypertrophy of the retinal pigment epithelium, lipomas, epidermoid cysts and upper gastrointestinal polyps.

To date, more than 300 different *APC* gene mutations are recognized as the cause of FAP. Most of these mutations (insertions, deletions, nonsense mutations, etc.) cause a truncated or inactive protein [58]. *APC* mutations have been subsequently found in ~80% of sporadic colorectal tumors, confirming that APC acts as a central gatekeeper protein in colorectal tumorigenesis [90].

Inherited or somatic mutations that inactivate or destroy APC function prevent effective degradation of β-catenin, promoting the aberrant activation of canonical Wnt signaling. This leads to the development of non-invasive colonic adenomas (polyps) because β-catenin nuclear accumulation causes the overexpression of growth-promoting genes [91]. The same outcome can arise through mutations in *CTNNB1* and *AXIN2*, though these are significantly less frequent than mutations in APC [92].

### Epigenetic modifications affecting the Wnt pathway

In addition to the genetic mutations, epigenetic modifications can contribute to aberrant activation of the canonical Wnt pathway. This can occur at various levels and determines the silencing or promoting of specific genes. In particular, aberrant methylation of CpG islands within gene promoter regions represents one of the most studied mechanisms of gene silencing and it is associated with selective transcriptional inactivation.

Many evidences indicate, for example, that almost complete loss of SFRPs at the protein levels are frequently correlated with gene promoter hypermethylation in several pathologies such as colon carcinomas, hepatocarcinomas [93], prostate cancer, human brain cancers [94], non-small cell lung cancer [95], esophageal carcinoma [96], myeloproliferative neoplasms.

A loss of SFRP expression, through epigenetic silencing, contributes to the constitutive activation of autocrine Wnt signaling affecting cell proliferation and potentially enhancing the cell growth and promoting malignant transformation and cancer cell survival [1,2,97,98].

### Wnt pathway’s interaction with the microenvironment

Regulators of the microenvironment, such as the extracellular matrix, growth factors and inflammatory factors, are associated with the aberrant activation of Wnt pathway and the promotion of several diseases.

#### Inflammation and Wnt pathway signaling

Inflammation is a critical defense mechanism against various harmful stimuli, although aberrant regulation may lead to diseases. The inflammation process, caused by injury, leads to wound healing, tissue repair and regeneration. Damaged epithelial and endothelial cells release inflammatory factors, growth factors, cytokines, and chemokines, which subsequently initiate an influx of neutrophils and monocytes to the site of the damaged tissue. Macrophages secrete platelet-derived growth factor (PDGF), connective tissue growth factor (CTGF) and transforming growth factor-β (TGF-β). They also activate and convert fibroblasts into myofibroblasts, which are engaged in extracellular matrix (ECM) deposition and scar formation [99,100]. Cytokines activate gene transcription regulators that are involved in stem cell renewal and proliferation, critical for tissue repair [100-103]. In case of repetitive injuries or unresolved damage, the inflammatory process can lead to aberrant fibroblast activation and excessive ECM accumulation with subsequent tissue fibrosis that can evolve into fibrotic disease and potential tumor initiation [100,101,104].

GSK3 has a crucial role in inflammation because it promotes pro-inflammatory cytokine production (IL-6, IL-1β and TNF-α) and cell migration [71,105,106]. Furthermore, two major pro-inflammatory cytokines, IFNγ and TNFα, are key regulators of β-catenin signaling and the most highly induced mediators in the inflamed tissue [107]. Thus there is a lasting involvement of Wnt/β-catenin signaling during the inflammation process that is associated with pathogenic disorder.

#### Wnt pathway and fibro-proliferative diseases

The development and progression of several fibrotic diseases is the outcome of the Wnt pathway cross-talk with other signaling pathways and pro-inflammatory mediators [108].

In general, aberrant Wnt/β-catenin signaling activation drives fibrogenesis through interaction with profibrotic growth factors, epithelial cell transformation, myofibroblasts activation and proliferation [109]. Mutant mice models demonstrate the involvement of β-catenin signaling in fibroproliferative diseases [110,111]. Furthermore, in fibrotic diseases, Wnts and positive regulators of β-catenin are upregulated, whereas inhibitors of Wnt/β-catenin signaling are downregulated [110].

The cross-talk between Wnt/β-catenin and TGF-β pathways has been demonstrated in several fibroproliferative disorders such as Dupuytren’s disease and pulmonary fibrosis [112,113]. TGF-β regulates the fibroblast activation to myofibroblast [81,108]. Wei et al. showed that Wnt3a activates the TGF-β cascade inducing the expression of pro-fibrotic genes [81,108]. On the other hand, TGF-β signaling seems to up-regulate Wnt/β-catenin pathway by decreasing the expression of Dkk-1, which in turn, inhibits the canonical Wnt pathway [81].

## Desmoid-type fibromatosis: A pathology arising from Wnt pathway genetic alteration and microenvironmental factors

Desmoid-type fibromatosis (DFs) can be an example of pathology arising from direct Wnt/β-catenin signaling alteration (Wnt mediator mutations) as well as indirect Wnt deregulation by involvement of the microenvironment. DF is a rare myofibroblastic neoplasm arising from a defect in connective tissue regulation, the neoplasia is unable to metastasize but it shows marked local aggression and a high recurrence rate. Some DFs are consequence of local trauma including pregnancies and surgical treatments [114,115]; repeated injuries also might increase the risk of DF recurrence.

### Genetic cause of desmoid-type fibromatosis: mutations of *CTNNB1* gene

**Figure 2:**
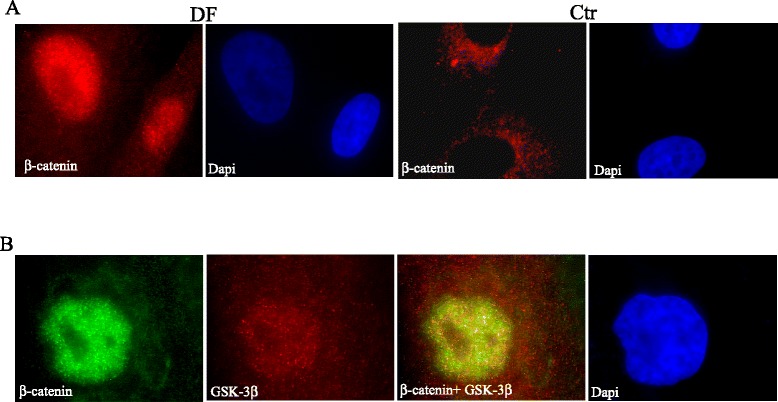
**Nuclear localization of GSK-3β and β-catenin in desmoid-type fibromatosis (DF) cells. A)** DF cells and control cells (ctr) were immunostained with anti-β-catenin (red). The nucleus was stained with DAPI (blue). The pictures show the nuclear localization of β-catenin in DF cells, and cytoplasmic staining in control cells. **B)** DF cells were immunostained with anti-β-catenin (green) and GSK-3β (red) antibodies. The nucleus was stained with DAPI. The merged picture shows colocalization of β-catenin and GSK-3β.

Desmoid-type fibromatosis might be one of the manifestations of the *APC* gene linked FAP but they are generally sporadic tumors. A range from 50% to 87% of sporadic DFs are characterized by mutations in codons 41 and 45 of exon 3 (p.Thr41Ala, p.Ser45Phe, and p.Ser45Pro) of the gene encoding β-catenin, *CTNNB1.* These codons are the serine and threonine phosphorylation sites required for β-catenin degradation [116,117]. Therefore, these mutations make phosphorylation impossible and promote β-catenin nuclear translocation [118]. A high level of nuclear β-catenin staining is the conventional diagnostic marker for DFs. Nuclear β-catenin is detected in almost 90% of the desmoid cells (Figure 2A). However, the abnormal expression of β-catenin is independent of *CTNNB1* mutations, suggesting that other factors might be involved in the alteration of the Wnt/β-catenin pathway in DFs. Intriguingly, in all DF cells, we have also noticed a very marked increase in nuclear GSK3β (95%) associated to β-catenin, suggesting that other changes involving the multiprotein complex are involved with the disease (Figure 2B) [118]. In addition, Caspi *et al.* demonstrated that GSK3β may have a nuclear function that impairs the Wnt pathway by a mechanism that does not involve phosphorylation and degradation of β-catenin [119,120]. These results support the potential significance of nuclear GSK3β as an additional marker for DF cells [118].

### Microenvironmental origin of desmoid-type fibromatosis

Nuclear accumulation of β-catenin in DFs can be also caused by microenvironmental factors such as inflammation, growth factors or hormones. Immunohistochemistry studies demonstrated that EGF, TGF-β, TNF-α, VEGF, phosphorylated SMAD2/3, COX2, and androgen receptor were significantly increased in desmoid tumors compared with healing scar tissue and quiescent fibrous tissue [121-124].

TGF-β is a modulator of β-catenin levels. Cultured fibroblasts, stimulated with TGF-β, induced nuclear accumulation of β-catenin and increased the activity of TCF/LEF reporter and transcription of the target gene *AXIN2* [81,108]. Expression of TGF-β-related cytokines has also been described in desmoid tumors [121,123-125].

Human DF samples also showed expression of the PDGFα and PDGFRα, metalloproteinases, ADAM12 and MMP2, and midkine, heparin-binding growth factor [126,127]. Expression of progesteron receptors has been reported in DFs samples, while they were negative for the estrogen receptor alpha [128,129].

## Perspectives of therapeutic approaches

### Wnt pathway inhibitors

As the canonical Wnt signaling is one of the central profibrotic signaling pathways [130] its inhibition on different levels (from ligand secretion to intracellular mediators) might be an effective antifibrotic treatment. Overexpression of the endogenous inhibitor Dkk-1 strongly ameliorated fibrosis in *in vitro* models mimicking early or later stages of human disease [108]. Thus it may be an attractive target for treating fibrosis, microvascular inflammation, tubule injury, and microvascular rarefaction [131]. However, the most effective therapy would be targeted the downstream complex in the pathway by using TCF/β-catenin antagonist that inhibits protein-protein interaction between TCF and β-catenin. Beyer and collaborators evaluated the antifibrotic effects of two small molecules, PKF118-310 and ICG-001, in the inflammatory model of bleomycin-induced dermal fibrosis. While PKF118-310 inhibits the β-catenin/TCF interaction, ICG-001 interferes with the recruitment of co-activators to β-catenin. The treatment with PKF118-310 and ICG-001 effectively inhibited canonical Wnt signaling reducing mRNA expression of Axin-2 (Figure 1B) [130]. These compounds prevent and reverse inflammation-driven fibrosis and reduce TGF-β dependent fibrosis.

Another mechanism for decreasing canonical Wnt signalling is to target the PDZ domain of DVL. Three compounds (NSC 668036, FJ9 and 3289–8625) have been identified to *in vitro* inhibit the Frizzled receptor-PDZ domain interaction*.*

Furthermore, the level of Axin in the β-catenin destruction complex is controlled by tankyrases, members of the PARP-family of poly-ADP-ribosylation enzymes. Small molecules, inhibiting the tankyrase 1 and tankyrase 2 enzymes, stabilize the level of Axin and promote the phosphorylation-dependent degradation of β-catenin by increasing the activity of the destruction complex [132]. Among these molecules Wang and collaborators demonstrated that XAV939 significantly inhibited the activation of Wnt/β-catenin signalling and attenuated bleomycin-induced lung fibrosis in mice [133]. The reduction of Wnt/β-catenin signaling, by the tankyrase inhibitors G007-LK and G244-LM, has been also demonstrated in APC mutant colorectal cancer (CRC) cell lines [134]. However, the clinical use of these inhibitors may be limited by the intestinal toxicity in APC-mutant CRC models and local or systemic toxicity in the fibrotic tissue of systemic sclerosis [134]. Intriguing, the pharmacological manipulation of Wnt pathway, using GSK3β inhibitors (lithium chloride, SB216763) (Figure 1A), is a promising therapeutic approach for several pathologies such as diabetes, stroke, mood disorders, inflammation, and Alzheimer’s disease [135].

Moreover, as GSK3 is a vital factor in inflammatory processes, inhibitors of GSK3 provide strong anti-inflammatory protection. GSK3 inhibitors were reported to reduce the inflammatory response in induced colitis in rats, as well as in arthritis and peritonitis in mice highlighting the potential therapeutic treatments in pathological conditions associated to inflammation [71,136,137].

### Therapeutic treatments described in desmoid-type fibromatosis

DF treatment is complicated by its recurrence, invasiveness, and persistence. Due to the heterogeneity of the desmoid-type fibromatosis and to the unpredictable clinical course, at the moment, the treatment is given on a case-by-case multimodal basis [138-140]. For this reason and for the absence of metastatic potential the “wait and see approach” is preferred when the tumors are asymptomatic and not located in area that could lead to functional limitations [141]. On the other hand, when the tumour mass causes discomfort, affects the function of involved organs or causes severe cosmetic damage, surgery is the preferred option, in association with radiotherapy and/or chemotherapy [142-144]. When the tumors are unresectable, radiotherapy is recommended [145,146]. For abdominal tumors, systemic therapy with non steroidal anti-inflammatory drugs, hormonal or biological agents, and cytotoxic drugs, is suggested. Different drugs have been used in clinics with different outcomes including Tamoxifen, Interferon-α, Doxorubicin, Imatinib and Sorafenib [146-150]. In particular, Imatinib mesylate has been reported to inhibit receptor tyrosine kinases, including PDGFR-α and PDGFR-β, as well as c-kit [151]. As desmoid tumor cells produce high levels of TGF-β, Toremifene which is an antiestrogen that inhibits collagen and TGF-β synthesis, has been used for *in vitro* desmoid cells. The results showed the reduction of receptor number only in desmoid cells, suggesting that Toremifene may reduce TGF-β's affinity for its receptors [121,152]. Toremifene also modifies the ECM components that regulate cytokine activity and cell migration.

An experimental animal model demonstrated that Apc(+)/Apc(1638 N) mice treated with Triparanol, an inhibitor of Hedgehog (Hh) signaling, develop few and smaller desmoid tumors compared with the untreated mice [153]. These data provide functional evidence that Hh pathway, associated with aberrant Wnt pathway, plays a key role in the maintenance of normal cells as the modulation of this pathway influences desmoid tumor behaviour. It also suggests Hh blockade as a therapeutic approach for this tumor type [153]. Hyperthermic isolated limb perfusion with TNF-α and Melphalan resulted to be an effective treatment in desmoid tumor recurrence of the limb or where resection threatens loss of function [154-156].

## Conclusions

The Wnt/β-catenin pathway is a great example of heavily context dependent cellular pathways with several ligands, receptors, transmitters and modulators. In general the interactions of the Wnt/β-catenin pathway with the other cellular processes clearly state its importance for the cell and the entire organism.

The most direct therapeutic approach against the deregulation of the Wnt/β-catenin pathway is to target the components of the pathways themselves or their closest interactors.

The dependence of the Wnt/β-catenin pathway on its microenvironment can be exploited as a potential target for therapeutic approaches in particular the host’s response to pathological cells including inflammation and the various growth factors produced in the attempt to “heal” the organism. This approach could also lead to a faster individuation of a valid treatment as several compounds are already commercialized, even if they are developed for completely unrelated diseases.

In synthesis, we need to continue studying on two fronts in order to find effective treatments for Wnt/β-catenin pathway related pathologies: the Wnt/β-catenin pathway itself and its role in the network comprising other pathways associated to the microenvironment.

## Consent

An informed written consent was obtained from the persons whose cell culture images were included in this review.

## Abbreviations

ADAM: Disintegrin and metalloproteinase domain-containing protein
APC: Adenomatous polyposis coli
β-Trcp: β-transducin-repeat-containing protein
BMP: Bone morphogenetic protein
CAMKII: Calmodulin mediated kinase II
CK1: Casein kinase 1
COX: Cytochrome c oxidase
CtBP: C-terminal-binding protein
CTD: C-terminal domain
CTGF: Connective tissue growth factor
CTNNB1: Catenin beta-1
DF: Desmoid-type fibromatosis
DKK-1: Dickkopf related protein 1
Dvl: Dishevelled
ECM: Extracellular matrix
EGF: Epidermal growth factor
FAP: Familial adenomatous polyposis
Fzd: Frizzled receptor
GBP/Frat: GSK3 binding protein/Frat
GSK3: Glycogen synthase kinase 3
Hh: Hedgehog
IFN-γ: Interferon gamma
IL: Interleukin
LDRL: Low-density lipoprotein repeats
LRP: Low-density lipoprotein receptor related protein
MAPK/ERK: Mitogen-activated protein kinase/extracellular regulated MAP kinase
MMP: Matrix metalloproteinase
mTOR: Mammalian target of rapamycin
PCP: Planar cell polarity
PDGF: Platelet-derived growth factor
PDGFR: Platelet-derived growth factor receptor
PI3K/Akt: Phosphotidylinositol 3 kinase/serine/threonine-protein kinase
PKC: Protein kinase C
PLC: Phospholipase C
PP1: Protein phosphatase 1
PP2A: Protein phosphatase 2A
sFRP: Secreted frizzled related-protein
SMAD: Small mothers against decapentaplegic
TCF/LEF: T cell factor/lymphoid enhancer factor
TGF-β: Transforming growth factor β
TNF-α: Tumor necrosis factor-α
VEGF: Vascular endothelial growth factor
WIF: Wnt inhibitor protein
